# Post-TAVR Neo-Sinus Geometry and Impaired Ventricular Recovery Are Associated with Early Leaflet Thrombosis: First MDCT Insights from Vietnam

**DOI:** 10.3390/jcm15166219

**Published:** 2026-08-11

**Authors:** Phi Dinh Truong, Hoai Thi Thu Nguyen, Quang Ngoc Nguyen, Linh Huynh Dinh, Nguyet Minh Thi Giap, Thai Quoc Nguyen, Minh Nhat Pham, Than Xuan Le, Long Phi Ngo, Trang Ngoc Nguyen, Hue Minh Thi Bui, Thanh Van Nguyen, Olivier Morel, Hung Manh Pham

**Affiliations:** 1 Vietnam National Heart Institute, Bach Mai Hospital, Hanoi 11520, Vietnam; hoainguyen1973@gmail.com (H.T.T.N.); dinhhuynhlinh@gmail.com (L.H.D.); gminhnguyet1979@gmail.com (N.M.T.G.); drnguyenquocthai@gmail.com (T.Q.N.); pnminhtm@gmail.com (M.N.P.); le.xuan.than.yhn@gmail.com (T.X.L.); drlongcvs.vnhi@gmail.com (L.P.N.); bsthanhnguyenvan@gmail.com (T.V.N.); 2 Department of Cardiology, Hanoi Medical University, Hanoi 11520, Vietnam; quangtm@gmail.com (Q.N.N.); hungmpham@gmail.com (H.M.P.); 3 Department of Internal Medicine, VNU University of Medicine and Pharmacy, Hanoi 11314, Vietnam; 4 Vinmec Times City, Vinmec International Hospital, Hanoi 11622, Vietnam; 5 Institute of Diagnostic and Interventional Radiology, Bach Mai Hospital, Hanoi 11520, Vietnam; drnguyenngoctrang@gmail.com; 6 Department of Anesthesiology and Critical Care, Hanoi Medical University, Hanoi 11520, Vietnam; buiminhhue1980@gmail.com; 7 Department of Cardiology, University Hospital of Strasbourg, 67098 Strasbourg, France; 8 Research Unit—UR3074—Translational Cardiovascular Medicine, University of Strasbourg, 67000 Strasbourg, France; 9 GERCA (Groupe pour l’Enseignement et la Recherche Cardiologique en Alsace), 67200 Strasbourg, France

**Keywords:** transcatheter aortic valve replacement, multidetector computed tomography, hypoattenuated leaflet thickening, leaflet thrombosis

## Abstract

**Background/Objectives:** Leaflet thrombosis after transcatheter aortic valve replacement (TAVR), typically identified as hypoattenuated leaflet thickening (HALT) on multidetector computed tomography (MDCT), is increasingly recognized as an early manifestation of bioprosthetic valve dysfunction. However, factors associated with early HALT in Southeast Asian populations remain poorly characterized. The objective of this study was to characterize MDCT features of leaflet thrombosis and identify factors independently associated with early HALT after TAVR. **Methods:** In this two-center observational study, patients undergoing MDCT 1–3 months after TAVR were evaluated. Leaflet thrombosis was defined as HALT. Leaflet involvement, thrombus severity, anatomical distribution, and post-implantation geometric parameters were analyzed. Multivariable logistic regression identified factors associated with early HALT. **Results:** Among 65 patients, HALT was detected in 30.8%, predominantly as subclinical leaflet thrombosis (29.2%), whereas clinical valve thrombosis was rare (1.5%). Most cases involved one or two leaflets, with preferential involvement of the non-coronary cusp. Patients with HALT showed greater increases in transvalvular peak gradients, and a peak gradient increase ≥10 mmHg was more frequent than in patients without HALT (30.0% vs. 4.4%; *p* = 0.008). Severe RCA ostial eccentricity (grade 3–4) combined with a neo-sinus–RCA distance > 13 mm was independently associated with HALT (adjusted OR 5.52; *p* = 0.006). Lack of left ventricular ejection fraction improvement after TAVR was also independently associated with HALT (adjusted OR 4.44; *p* = 0.018), though given the limited number of HALT events (*n* = 20), these multivariable associations should be regarded as exploratory and hypothesis-generating. **Conclusions:** Early post-TAVR leaflet thrombosis is common and predominantly subclinical. CT-derived neo-sinus geometry and impaired ventricular recovery were independently associated with HALT, supporting the hypothesis that altered neo-sinus washout contributes to thrombus formation.

## 1. Introduction

Transcatheter aortic valve replacement (TAVR) has become an established treatment for severe aortic stenosis across a broad spectrum of surgical risk, leading to growing attention to complications related to bioprosthetic valve durability, particularly leaflet thrombosis [1,2]. With the increasing use of multidetector computed tomography (MDCT), hypoattenuated leaflet thickening (HALT) has been reported in 10% to 40% of patients [1]. This entity may impair leaflet motion, adversely affect transvalvular hemodynamics, and it is increasingly recognized as a potential precursor of thromboembolic events and late bioprosthetic valve dysfunction.

The mechanisms underlying leaflet thrombosis after TAVR remain incompletely understood. Among the proposed contributors, post-implantation geometric factors have gained particular interest, as they may promote flow stagnation and low shear stress conditions that favor thrombus formation [1]. In this context, anatomical differences between populations may be relevant. Patients from Southeast Asia, including Vietnam, often present with smaller aortic annulus dimensions compared with Western populations, a feature increasingly recognized as relevant to TAVR practice in Asia [3]. Small annulus size has been suggested as a potential factor associated with leaflet thrombosis, particularly when intra-annular prostheses are used, due to altered flow dynamics and increased leaflet interaction within a constrained neo-sinus.

In addition to geometric considerations, the interaction between the transcatheter valve and the native calcified leaflets may also contribute to thrombogenesis. The native valve tissue, which is not excised during TAVR, may act as a reservoir of bioactive and prothrombotic factors, further promoting local thrombus formation in the setting of altered flow conditions [4].

Despite these hypotheses, existing evidence remains inconsistent, and the relative contribution of anatomical, procedural, and biological factors is still debated. Despite the growing adoption of TAVR across the Asia-Pacific region, region-specific evidence remains limited, particularly in Southeast Asia [5], and to our knowledge, no study from Vietnam has specifically addressed this issue.

Therefore, we conducted a two-center study to characterize the features of leaflet thrombosis on MDCT and to investigate factors associated with early HALT in patients undergoing TAVR during short-term follow-up (1–3 months after the procedure).

## 2. Materials and Methods

### 2.1. Study Design and Setting

This study was designed as a two-center observational longitudinal study with both retrospective and prospective components, in accordance with the STROBE recommendations. The study was conducted at the Vietnam National Heart Institute—Bach Mai Hospital and Hanoi Medical University Hospital, tertiary referral centers for structural heart interventions in Vietnam.

### 2.2. Participants

All consecutive patients with severe aortic stenosis who underwent transcatheter aortic valve replacement (TAVR) at the Vietnam National Heart Institute–Bach Mai Hospital and Hanoi Medical University Hospital during the study period were screened for eligibility. Patients who survived to the scheduled follow-up and had no contraindications to contrast-enhanced multidetector computed tomography (MDCT) were invited to undergo post-TAVR MDCT.

Inclusion criteria were successful TAVR, written informed consent, and completion of the scheduled 1–3-month follow-up evaluation, including clinical assessment, laboratory testing, transthoracic echocardiography, and contrast-enhanced MDCT.

Patients were excluded if they died or required surgical conversion before the scheduled follow-up, had contraindications to contrast-enhanced MDCT (e.g., severe renal dysfunction), declined follow-up MDCT, or underwent MDCT outside the prespecified follow-up window or with insufficient image quality for analysis.

Of the 97 consecutive patients screened, 65 were included in the final analysis. The patient selection process is summarized in Supplementary Figure S1.

### 2.3. Study Size

All consecutive eligible patients during the predefined study period were included. Because this was an exploratory observational study and reliable prior estimates of the frequency of early HALT and the effect sizes associated with post-TAVR neo-sinus geometric parameters in Southeast Asian populations were unavailable, a formal a priori sample-size calculation was not feasible.

### 2.4. Study Period

Patients were enrolled between August 2023 and May 2026, with follow-up assessments performed at 1–3 months after TAVR.

### 2.5. Variables and Definitions

The primary outcome was leaflet thrombosis detected on MDCT, defined by the presence of hypoattenuated leaflet thickening (HALT). Valve thrombosis was further classified as subclinical leaflet thrombosis (SLT) or clinical valve thrombosis (CVT) according to the VARC-3 definitions [6]. SLT was defined as HALT, with or without reduced leaflet motion (RLM), in the absence of clinically relevant manifestations or significant hemodynamic deterioration. CVT was defined as imaging-confirmed valve thrombosis associated with clinical manifestations and/or hemodynamic valve deterioration.

Secondary variables included: the extent and location of leaflet involvement, HALT severity, the number of affected leaflets, RLM, and transvalvular hemodynamic parameters.

Exposure variables included: baseline clinical characteristics and cardiovascular risk factors, pre-TAVR anatomical features, discharge antithrombotic therapy, and post-procedural geometric parameters, including valve position, coronary ostial eccentricity, prosthesis expansion, and neo-sinus-related measurements.

### 2.6. Data Sources and Follow-Up Assessment

Clinical, imaging, and procedural data were obtained from hospital medical records and prospectively collected follow-up visits. All patients underwent standardized evaluation including: clinical assessment, laboratory testing, transthoracic echocardiography, and contrast-enhanced MDCT at 1–3 months after TAVR.

### 2.7. MDCT Acquisition Protocol

Post-TAVR MDCT was performed using a second-generation dual-source CT scanner with two detector arrays (Somatom Definition Flash, Siemens Healthcare, Forchheim, Germany). Retrospective ECG-gated contrast-enhanced acquisition was performed in the craniocaudal direction with a temporal resolution of 75 ms. Tube voltage and tube current were automatically adjusted using the Siemens Care kV and Care Dose systems. A total of 50–70 mL of iodinated contrast medium containing 400 mg iodine/mL (Imeron 400, Bracco, Konstanz, Germany) was administered intravenously at 4–5 mL/s. Images were reconstructed every 5% of the RR interval from 20% to 100% of the cardiac cycle, with a slice thickness of <1 mm using the dedicated B46f stent reconstruction kernel. Multiplanar, cine, and volume-rendered reconstructions were analyzed using Syngo Multimodality Workplace (Siemens Healthcare, Forchheim, Germany).

### 2.8. MDCT Image Interpretation and Geometric Measurements

MDCT datasets were independently analyzed by two experienced cardiovascular imaging specialists blinded to clinical outcomes and follow-up echocardiographic findings. Disagreements were resolved by third-reader adjudication.

HALT was identified visually on contrast-enhanced multiplanar images according to VARC-3 definitions [6], without a predefined Hounsfield-unit threshold. It was primarily assessed during diastole and graded as <25%, 25–50%, 50–75%, or >75% leaflet involvement. RLM was primarily assessed during systole using multiphase cine reconstructions and graded using the same percentage categories. Final classifications were based on the cardiac phases providing optimal leaflet visualization.

Additional measurements included leaflet-specific involvement, maximum HALT thickness, prosthesis expansion, valve position, coronary ostial eccentricity, and neo-sinus-to-coronary distances. Measurement methods are illustrated in Supplementary Figures S2 and S3.

### 2.9. Echocardiographic Assessment

Transthoracic echocardiography was performed before TAVR, before hospital discharge, and at the scheduled 1–3-month follow-up by experienced echocardiographers according to current recommendations. Left ventricular ejection fraction (LVEF) was measured using the biplane Simpson’s method. The change in LVEF (ΔLVEF) was calculated as the difference between follow-up and baseline LVEF, and no improvement in LVEF was defined as ΔLVEF ≤ 0 percentage points. Standard hemodynamic parameters, including peak and mean transvalvular gradients, were also recorded. Measurements were not averaged across readers.

### 2.10. Bias

Consecutive screening, predefined eligibility criteria, standardized imaging protocols, blinded independent MDCT interpretation, and third-reader adjudication were used to reduce selection and measurement bias. Nevertheless, the analyzed cohort comprised patients who survived to follow-up, remained eligible for contrast-enhanced MDCT, and had interpretable examinations. Selection and survivor bias therefore cannot be excluded.

### 2.11. Statistical Methods

Statistical analyses were performed using SPSS version 23.0 (IBM Corp., Armonk, NY, USA). Categorical variables are presented as counts and percentages, and continuous variables as mean ± standard deviation or median (interquartile range). Between-group comparisons used the Mann–Whitney U test, Chi-square or Fisher’s exact test, and Kruskal–Wallis test, as appropriate.

Variables associated with HALT were identified by univariable logistic regression; continuous variables showing significant or near-significant associations were dichotomized using Youden-index-derived ROC cutoffs before entry into the multivariable model. Linearity of continuous predictors with the log odds of HALT was assessed using the Box–Tidwell procedure and restricted cubic splines. Variables with *p* < 0.10 in univariable analysis or judged clinically relevant were considered for the multivariable model, with the number of candidate variables limited according to the number of observed outcome events to minimize the risk of overfitting. A two-sided *p*-value < 0.05 was considered statistically significant.

### 2.12. Use of Artificial Intelligence in Manuscript Preparation

The authors used ChatGPT (GPT-5.5, Plus version; OpenAI, San Francisco, CA, USA) to assist in generating illustrative figures, schematic diagrams, and data-based charts, and in editing English sentence structure. ChatGPT was not used for data analysis, interpretation, or scientific content generation. All outputs were reviewed and verified by the authors, who take full responsibility for the manuscript’s accuracy.

## 3. Results

### 3.1. Characteristics of Aortic Leaflet Thrombosis at 1–3 Months After TAVR

Baseline clinical and paraclinical characteristics of the study population are summarized in Table 1 (see Supplementary Table S10 for further details). The mean interval between transcatheter aortic valve replacement (TAVR) and multidetector computed tomography (MDCT) assessment was 41.6 ± 14.4 days, corresponding to an early post-procedural evaluation period.

Among the 65 patients included, leaflet thrombosis (HALT) was identified in 20 cases (30.8%). The vast majority of cases were subclinical leaflet thrombosis (SLT), observed in 19 patients (29.2%), whereas clinical valve thrombosis (CVT) was detected in only 1 patient (1.5%) (Supplementary Table S1). Overall, SLT accounted for 95% of thrombosis cases, confirming that early post-TAVR thrombosis is predominantly subclinical.

Regarding leaflet involvement, thrombosis most frequently affected a single leaflet (11/20, 55%), followed by two leaflets (7/20, 35%) and three leaflets (2/20, 10%). In terms of anatomical distribution, the non-coronary leaflet was most commonly involved (13/20, 65%), followed by the left coronary leaflet (11/20, 55%) and right coronary leaflet (7/20, 35%) (Supplementary Tables S2–S4). As multiple leaflets could be affected simultaneously, cumulative percentages exceeded 100%.

Thrombosis severity was categorized into four grades (<25%, 25–50%, 50–75%, and >75%), with the following distribution: grade 1 in 1 case (5%), grade 2 in 7 cases (35%), grade 3 in 8 cases (40%), and grade 4 in 4 cases (20%). Grade 3 thrombosis was the most common presentation. The mean maximal thrombosis thickness was 5.68 ± 1.99 mm. (Supplementary Tables S5 and S6).

Reduced leaflet motion (RLM) was observed in 16 of 20 cases (80%). Among these, RLM severity was graded as mild (<25%) in 4 cases (25%), moderate (25–50%) in 7 cases (43.8%), and moderately severe (50–75%) in 5 cases (31.2%); no cases of severe RLM (>75%) were identified. RLM most frequently involved the non-coronary leaflet, either in isolation or in combination with other leaflets (Supplementary Tables S7–S9).

### 3.2. Impact of Leaflet Thrombosis on Aortic Valve Hemodynamics at 1–3 Months After TAVR

Clinical valve thrombosis (CVT) was associated with increased transvalvular gradients, whereas isolated hypoattenuated leaflet thickening (HALT) generally remained subclinical and was not associated with marked early hemodynamic deterioration.

**Figure 1:**
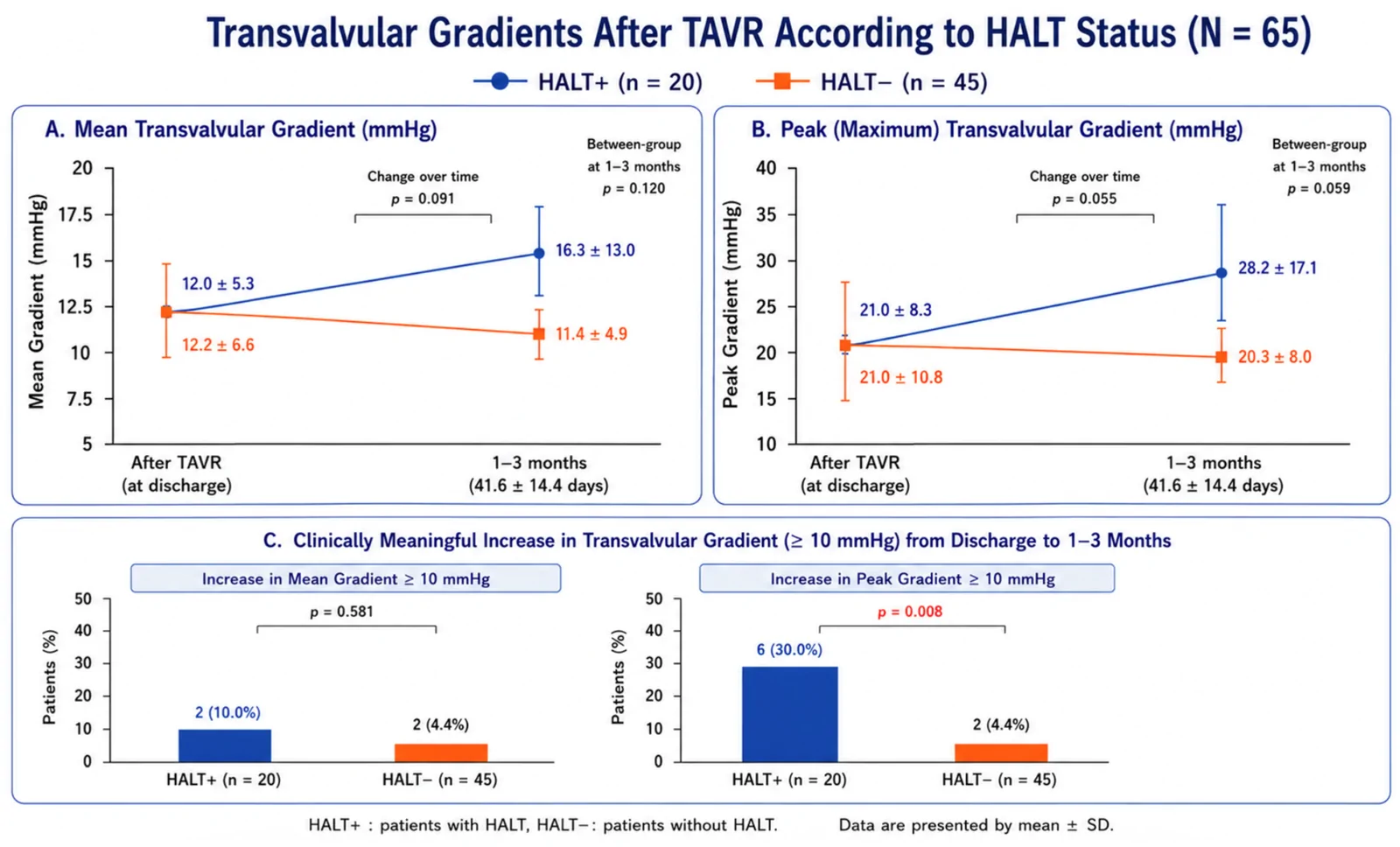
Impact of HALT on aortic transvalvular hemodynamics at 1–3 months after TAVR.

Immediately after TAVR, mean transvalvular gradients were similar between patients with and without HALT (12.0 ± 5.3 mmHg vs. 12.2 ± 6.6 mmHg; *p* = 0.873). At 1–3 months, mean gradients tended to be higher in the HALT group (16.3 ± 13.0 mmHg vs. 11.4 ± 4.9 mmHg), although the difference did not reach statistical significance (*p* = 0.120). Likewise, the change in mean gradient over time was numerically greater among patients with HALT (+4.3 ± 12.4 mmHg vs. −0.8 ± 5.5 mmHg; *p* = 0.091) (Figure 1).

Peak transvalvular gradients immediately after TAVR were also comparable between groups (21.0 ± 8.3 mmHg vs. 21.0 ± 10.8 mmHg; *p* = 0.983). However, at follow-up, patients with HALT demonstrated a trend toward higher peak gradients (28.2 ± 17.1 mmHg vs. 20.3 ± 8.0 mmHg; *p* = 0.059). Similarly, the increase in peak gradient during follow-up was greater in the HALT group (+7.3 ± 16.7 mmHg vs. −0.7 ± 8.6 mmHg; *p* = 0.055) (Figure 1).

Importantly, a clinically meaningful increase in peak transvalvular gradient (≥10 mmHg) was significantly more frequent among patients with HALT compared with those without thrombosis (30.0% vs. 4.4%; *p* = 0.008), whereas an increase in mean gradient ≥ 10 mmHg did not differ significantly between groups (10.0% vs. 4.4%; *p* = 0.581). Across the entire cohort, the incidence of an increase in peak transvalvular gradient (≥10 mmHg) was 12.3% (8/65) (Figure 1).

Overall, these findings suggest that most cases of isolated HALT remain hemodynamically mild during early follow-up after TAVR. Nevertheless, the observed trend toward progressive transvalvular gradient increase, particularly in cases with more advanced thrombotic involvement, although CVT was rare in this cohort, supports the concept that HALT and CVT may represent different stages within the continuum of bioprosthetic valve thrombosis and dysfunction. Even in the absence of overt early hemodynamic compromise, subtle leaflet thickening and motion abnormalities detected on CT may therefore warrant closer imaging surveillance after TAVR.

### 3.3. Factors Associated with Aortic Leaflet Thrombosis at 1–3 Months After TAVR

In univariable analysis, several echocardiographic, laboratory, and CT-derived parameters were associated with HALT formation during short-term follow-up after TAVR (Table 2; see Supplementary Table S11 for further details). For continuous variables, restricted cubic spline modeling confirmed no significant departure from linearity with the log odds of HALT (Supplementary Table S12). Patients with HALT had significantly higher peak transvalvular gradients at 1–3 months compared with those without HALT (28.2 ± 17.1 vs. 20.3 ± 8.0 mmHg; OR per 10-mmHg increment: 1.97, 95% CI: 1.10–3.39; *p* = 0.032). Similarly, a greater increase in peak gradient from discharge to 1–3 months was weakly associated with HALT (OR per 10-mmHg increment: 2.16, 95% CI: 1.00–4.41; *p* = 0.042). Importantly, an increase in peak gradient ≥10 mmHg was markedly more frequent in the HALT group (30.0% vs. 4.4%) and was strongly associated with leaflet thrombosis (OR: 9.21, 95% CI: 1.67–50.96; *p* = 0.008).

Regarding left ventricular functional recovery, patients with HALT demonstrated significantly less improvement in EF during follow-up. The change in EF from pre-TAVR to 1–3 months was substantially lower in the HALT group (1.3 ± 10.9 vs. 8.8 ± 9.5%; OR per 5-percentage-point increment: 0.66, 95% CI: 0.47–0.90; *p* = 0.012). Moreover, absence of EF improvement or a reduction in EF after TAVR was significantly associated with HALT (55.0% vs. 20.0%; OR: 4.89, 95% CI: 1.56–15.35; *p* = 0.008).

In addition, hemoglobin concentration at 1 month after TAVR was higher in patients with HALT (137.8 ± 14.0 vs. 128.6 ± 16.8 g/L) and was significantly associated with HALT risk (OR per 1 g/L increase: 1.04, 95% CI: 1.00–1.08; *p* = 0.042).

Among CT-derived geometric parameters, severe RCA ostial eccentricity (grade 3–4) was more common in the HALT group and was weakly associated with a higher risk of leaflet thrombosis (70.0% vs. 42.2%; OR: 3.19, 95% CI: 1.03–9.86; *p* = 0.043). For neo-sinus–RCA distance, exploratory ROC analysis showed modest discrimination for HALT (AUC 0.634, 95% CI: 0.482–0.787; *p* = 0.083), with a Youden-optimal cut-off of 13.37 mm that classified patients identically to the predefined 13 mm threshold used in this study (Supplementary Figure S4). Using this threshold, neo-sinus–RCA distance > 13 mm was significantly associated with HALT (75.0% vs. 44.4%; OR: 3.75, 95% CI: 1.16–12.09; *p* = 0.027). Notably, the combination of severe RCA ostial eccentricity (grade 3–4) and neo-sinus–RCA > 13 mm demonstrated an even stronger association with HALT (60.0% vs. 20.0%; OR: 6.00, 95% CI: 1.89–19.04; *p* = 0.002).

In multivariable analysis, the combination of severe RCA ostial eccentricity (grade 3–4) and neo-sinus − RCA distance > 13 mm remained independently associated with HALT (adjusted OR: 5.52, 95% CI: 1.63–18.73; *p* = 0.006). Additionally, absence of EF improvement or EF reduction at 1–3 months after TAVR also remained independently associated with HALT (adjusted OR: 4.44, 95% CI: 1.29–15.29; *p* = 0.018) (Table 3). Given the limited number of HALT events (*n* = 20), the multivariable model was restricted to two clinically relevant variables to minimize the risk of overfitting.

## 4. Discussion

The present study demonstrates that leaflet thrombosis is a frequent early finding after TAVR, affecting nearly one-third of patients, while remaining predominantly subclinical. The combination of severe RCA ostial eccentricity (grade 3–4) and increased neo-sinus–RCA distance was independently associated with leaflet thrombosis, highlighting the importance of post-implantation valve geometry in thrombus formation. Moreover, HALT was independently associated with absence of left ventricular ejection fraction recovery, suggesting a possible bidirectional relationship between impaired ventricular functional recovery and leaflet thrombosis, even in the absence of overt valve dysfunction.

In this study, the incidence of leaflet thrombosis (HALT) within the first 1–3 months after TAVR was 30.8%, predominantly driven by subclinical leaflet thrombosis (SLT) (29.2%), whereas clinical valve thrombosis (CVT) was rare. This incidence is consistent with prior reports, including the RESOLVE and SAVORY registries, which described HALT rates ranging from 10% to 40% depending on imaging timing and patient characteristics [1]. Similarly, meta-analytic data suggest that HALT may occur in up to one-third of patients following TAVR [7]. Overall, these findings reinforce that leaflet thrombosis is a relatively frequent phenomenon when systematically assessed using MDCT.

Current evidence on leaflet thrombosis after TAVR is largely derived from Western cohorts, whereas data from Asian populations remain limited. Available studies, predominantly from Japan, China, and South Korea, suggest that HALT is not uncommon, with reported incidences ranging from 9% to 30%, and most cases are subclinical [8,9,10,11,12,13,14,15]. However, its determinants and clinical implications in Asian patients remain incompletely characterized [8,9,10,11,12,13,14,15]. Data from Southeast Asia are particularly scarce. To our knowledge, this study is the first from Vietnam and among the few from Southeast Asia to systematically evaluate leaflet thrombosis using MDCT. Notably, our cohort exhibited several anatomical and procedural characteristics previously associated with an increased risk of leaflet thrombosis and impaired bioprosthetic valve durability [1,2,16], including a relatively small annulus (438.7 ± 113.4 mm^2^), severe valvular calcification (Agatston score 4136.8 ± 2425.4, with 70.8% severe calcification), and predominant use of balloon-expandable intra-annular valves (75.4%). These findings are particularly relevant in the Asian setting, where small annular anatomy is highly prevalent and frequently necessitates the use of small balloon-expandable prostheses [17,18]. Together with severe native valve calcification, these factors may adversely affect neo-sinus flow dynamics and valve hemodynamics, thereby predisposing to leaflet thrombosis and subsequent bioprosthetic valve dysfunction.

From a mechanistic perspective, the combination of a small annulus, extensive residual native valve calcification, and intra-annular valve design may promote unfavorable neo-sinus hemodynamics [19]. Reduced washout and increased blood stasis at the leaflet base may facilitate thrombus formation, while calcified native leaflets may further provide a local prothrombotic substrate [4,20]. Consistent with this pathophysiological framework, we observed a relatively high incidence of HALT (30.8%), at the upper range of previously reported rates. Collectively, these findings support the concept that leaflet thrombosis is a flow-mediated phenomenon and suggest that population-specific anatomical characteristics may contribute to its occurrence after TAVR.

We observed a non-uniform distribution of thrombosis across valve leaflets, with a predominance in the non-coronary, followed by left and right coronary leaflets. While prior studies have reported heterogeneous patterns [8,21], our findings suggest a preferential involvement of the non-coronary cusp. This pattern may be explained by local flow conditions within the neo-sinus. Unlike coronary-facing leaflets, which benefit from diastolic coronary flow washout, the non-coronary leaflet is more exposed to recirculation zones and flow stagnation, thereby favoring thrombus formation. The frequent involvement of multiple leaflets further suggests that thrombosis reflects global alterations in sinus hemodynamics rather than isolated leaflet-specific processes.

***With respect to hemodynamic consequences***, the hemodynamic significance of HALT remains controversial. Most CT-based studies and contemporary reviews suggest that HALT is frequently subclinical and is not consistently associated with increased transvalvular gradients or overt bioprosthetic valve dysfunction [1,22,23]. Nevertheless, our findings suggest a more nuanced relationship. Although hemodynamic impairment remained modest in most patients, HALT was associated with progressive transvalvular gradient elevation during early follow-up. While post-procedural hemodynamics were comparable between groups, patients with HALT exhibited greater temporal increases in transvalvular gradients, particularly peak gradients. Notably, a clinically meaningful increase in peak gradient ≥ 10 mmHg occurred significantly more frequently among patients with HALT (30.0% vs. 4.4%; *p* = 0.008). These observations suggest that, although many cases of HALT may remain hemodynamically silent, a subset of patients may already develop measurable functional consequences during the early phase of thrombotic valve dysfunction.

This interpretation is supported by recent studies from Hein et al. [24] and from Trimaille, Morel, and colleagues [25]. In the largest multicenter TAVR registry reported to date, including 7392 patients, early hemodynamic valve deterioration—defined as a ≥10-mmHg increase in mean transvalvular gradient within 3 months after TAVR—was independently associated with stroke, bioprosthetic valve deterioration, and valve failure [25]. Importantly, smaller annular dimensions emerged as an independent predictor of early hemodynamic valve deterioration, a finding particularly relevant to our cohort characterized by relatively small annular anatomy.

Taken together, these findings suggest that HALT should not be regarded as a uniformly benign imaging finding. Rather, HALT likely encompasses a spectrum ranging from hemodynamically silent leaflet thickening to early thrombotic valve dysfunction accompanied by progressive gradient elevation. The latter may represent an early stage of thrombotic valve dysfunction in a subset of patients, although progression along this continuum is variable and has not been conclusively established. Routine echocardiographic surveillance, complemented by MDCT when transvalvular gradients increase, may facilitate the identification of potentially reversible thrombotic valve dysfunction before irreversible fibrocalcific remodeling develops.

***Regarding associated factors,*** one of the most important findings of the present study was the identification of a close interplay between post-TAVR valve geometry, early hemodynamic changes, and the development of HALT. In univariable analysis, patients with HALT demonstrated significantly higher transvalvular gradients at 1–3 months, together with greater temporal increases in peak gradients over time.

A particularly noteworthy finding was the independent association between RCA-oriented neo-sinus geometry and HALT risk. Both severe RCA ostial eccentricity (grade 3–4) and a neo-sinus–RCA distance > 13 mm were significantly associated with leaflet thrombosis, while their combination demonstrated an even stronger association and remained independently associated with HALT, although the wide confidence interval around this combined estimate (95% CI: 1.63–18.73) reflects the relatively small number of HALT events and indicates limited precision, supporting a hypothesis-generating rather than confirmatory interpretation. To our knowledge, this is among the first studies to demonstrate a combined effect of coronary eccentricity and neo-sinus geometry on leaflet thrombosis after TAVR. These anatomical parameters should be interpreted as surrogate markers that may reflect local flow conditions rather than direct evidence of impaired washout, as direct flow measurements were not performed in this study. Severe RCA ostial eccentricity may impair neo-sinus washout, while a larger neo-sinus–RCA distance may further reduce coronary flow-mediated clearance, promoting localized blood stasis and thrombus formation (Supplementary Figure S5); this mechanism is biologically plausible but remains hypothetical. Our findings are consistent with computational and experimental studies highlighting the central role of altered neo-sinus micro-hemodynamics in post-TAVR leaflet thrombosis [26,27,28]. Furthermore, commissural misalignment—a related but anatomically distinct geometric concept—has previously been associated with subclinical leaflet thrombosis [29]. Our results extend this concept by suggesting that increased neo-sinus–RCA distance may further exacerbate flow stagnation within the RCA-oriented sinus.

Another notable observation was the association between HALT and impaired ventricular recovery. Patients with HALT demonstrated significantly less improvement in left ventricular ejection fraction, while absence of EF recovery or EF decline remained independently associated with thrombosis. Although causality cannot be established, these findings suggest a bidirectional relationship whereby early leaflet thrombosis may adversely affect reverse remodeling, whereas impaired ventricular recovery may reflect an underlying hemodynamic or thrombogenic milieu predisposing to HALT. Residual confounding by baseline myocardial characteristics, including pre-existing myocardial dysfunction or fibrosis, cannot be excluded. Given the paucity of available data, this observation should be considered hypothesis-generating.

Our cohort also included a high proportion of bicuspid aortic valves (56.9%), a feature frequently encountered in Asian TAVR practice and increasingly recognized as a distinctive characteristic of Asian TAVR populations [5]. Although bicuspid anatomy was not independently associated with HALT, its asymmetric geometry, elliptical annulus, and uneven calcification may contribute to altered neo-sinus flow, prosthesis underexpansion, and commissural misalignment. Consistent with this concept, Nagasaka et al. identified underexpansion and misalignment as important determinants of leaflet thrombosis and adverse outcomes following TAVR in bicuspid anatomy [30]. Given the limited sample size and small number of HALT events, our study was not adequately powered to test whether bicuspid anatomy modifies the association between coronary ostial eccentricity and HALT, and this interaction warrants confirmation in larger bicuspid-enriched cohorts. Similarly, given the predominant use of balloon-expandable valves (75.4%) in this cohort, these anatomical associations should be interpreted within the context of our study population and require validation across more diverse valve platforms.

Overall, these findings reinforce the concept that HALT is not merely an incidental imaging finding but rather the consequence of complex interactions between valve geometry, neo-sinus hemodynamics, and thrombotic remodeling processes. The identification of independent geometric factors may provide opportunities for individualized TAVR strategies, including optimization of valve positioning relative to the coronary ostia, minimization of neo-sinus flow stagnation, and targeted imaging surveillance in high-risk patients.

### Study Limitations

This study has several limitations. First, it was conducted at two centers with a relatively small sample size, which may limit statistical power and the generalizability of the findings. Because of the limited number of HALT events, the multivariable model should be considered exploratory and hypothesis-generating, and these associations require external validation in larger, independent cohorts before being incorporated into clinical decision-making. Second, the observational design precludes causal inference, and residual confounding cannot be excluded despite systematic data collection. Third, MDCT assessment was performed at a single early time point (1–3 months), which does not allow evaluation of the temporal evolution of HALT or its long-term clinical impact. Fourth, although standardized imaging protocols were used, and disagreements were resolved by third-reader adjudication, required in 5 of 65 cases, interobserver variability in MDCT interpretation was not formally assessed using quantitative measures such as kappa statistics or intraclass correlation coefficients; similarly, small changes in LVEF may fall within the inherent interobserver and intraobserver variability of echocardiographic assessment. Finally, the lack of long-term clinical outcomes including stroke, valve reintervention, or structural valve deterioration prevents direct assessment of the relationship between early leaflet thrombosis and subsequent bioprosthetic valve dysfunction.

## 5. Conclusions

Early leaflet thrombosis after TAVR is a common finding, predominantly manifesting as subclinical HALT. Thrombosis risk is independently associated with neo-sinus geometry—particularly RCA ostial eccentricity and increased neo-sinus–RCA distance—as well as limited early LVEF recovery. These findings support a multifactorial mechanism integrating valve–sinus geometry, local micro-hemodynamics, and early ventricular functional recovery. Although most HALT cases showed only modest early hemodynamic impairment, in a subset of patients, HALT may represent an early stage in the continuum toward bioprosthetic valve dysfunction. Given the absence of consensus supporting routine post-TAVR MDCT screening in asymptomatic patients, these findings instead support selective use of MDCT in patients with unfavorable valve geometry, unexplained hemodynamic deterioration, or clinically suspected leaflet thrombosis, alongside individualized post-TAVR management strategies.

## Figures and Tables

**Table 1:** Baseline Clinical and Paraclinical Characteristics of the Study Population.

Characteristics	Overall (*n* = 65)	HALT+ (*n* = 20)	HALT− (*n* = 45)	*p*
**Anthropometric measurements**				
Age (years)	74 (68–76)	74 (70–76)	73 (67–75)	0.343
Male sex	46 (70.8)	15 (75.0)	31 (68.9)	0.770
BMI (kg/m^2^)	22.4 (20.6–24.4)	20.9 (19.9–23.1)	22.7 (21.1–24.5)	0.088
BSA (m^2^)	1.65 (1.49–1.71)	1.61 (1.48–1.69)	1.66 (1.51–1.73)	0.313
**History**				
Type 2 diabetes mellitus	16 (24.6)	6 (30.0)	10 (22.2)	0.542
Hypertension	29 (44.6)	7 (35.0)	22 (48.9)	0.418
Dyslipidemia	10 (15.4)	2 (10.0)	8 (17.8)	0.711
Smoking	19 (29.2)	7 (35.0)	12 (26.7)	0.560
Coronary artery disease	12 (18.5)	5 (25.0)	7 (15.6)	0.490
Prior stroke	4 (6.2)	0 (0.0)	4 (8.9)	0.303
Peripheral artery disease	2 (3.1)	1 (5.0)	1 (2.2)	0.524
Chronic lung disease	6 (9.2)	2 (10.0)	4 (8.9)	1.000
Previous cardiac valve surgery	5 (7.7)	1 (5.0)	4 (8.9)	1.000
**Surgical risk**				
EuroSCORE II (%)	2.53 (1.42–5.33)	2.05 (1.35–4.78)	2.54 (1.44–5.33)	0.434
STS score (%)	2.63 (1.66–4.63)	2.97 (1.85–5.89)	2.56 (1.58–4.40)	0.345
**Pre-TAVR echocardiography**				
Pre-TAVR EF (%)	53.5 ± 13.5	56.2 ± 13.1	52.3 ± 13.7	0.283
Aortic valve area (cm^2^)	0.6 ± 0.2	0.6 ± 0.1	0.5 ± 0.2	0.598
Pre-TAVR peak gradient (mmHg)	87.2 ± 23.3	89.9 ± 19.7	86.1 ± 24.9	0.508
Pre-TAVR mean gradient (mmHg)	55.5 ± 15.8	56.9 ± 13.4	54.9 ± 16.9	0.612
Pre-TAVR Vmax (m/s)	4.6 ± 0.7	4.7 ± 0.7	4.6 ± 0.7	0.428
**Pre-TAVR MDCT characteristics**				
Tricuspid aortic valve	23 (35.4)	9 (45.0)	14 (31.1)	0.530
Bicuspid aortic valve	37 (56.9)	10 (50.0)	27 (60.0)	
Annular area (mm^2^)	438.7 ± 113.4	445.2 ± 107.4	435.8 ± 117.0	0.754
Left coronary artery height (mm)	14.1 ± 3.4	14.4 ± 2.5	14.0 ± 3.7	0.596
Right coronary artery height (mm)	16.4 ± 4.5	17.0 ± 5.9	16.1 ± 3.7	0.511
Annulus angle (degrees)	49.2 ± 9.9	51.2 ± 10.4	48.4 ± 9.7	0.305
Maximum ascending aorta diameter (mm)	38.3 ± 5.2	38.9 ± 4.8	38.1 ± 5.4	0.518
Calcification score (Agatston)	4136.8 ± 2425.4	4827.6 ± 2118.3	3829.8 ± 2524.6	0.126
Severe calcification	46 (70.8)	15 (75.0)	31 (68.9)	0.770
**TAVR procedure**				
Pre-dilatation	58 (89.2)	19 (95.0)	39 (86.7)	0.423
Balloon-expandable valve	49 (75.4)	17 (85.0)	32 (71.1)	0.375
Self-expandable valve	16 (24.6)	3 (15.0)	13 (28.9)	
Small THV size (20–23 mm)	30 (46.2)	10 (50.0)	20 (44.4)	0.410
Medium THV size (24.5–27.5 mm)	20 (30.8)	4 (20.0)	16 (35.6)	
Large THV size (29–30.5 mm)	15 (23.1)	6 (30.0)	9 (20.0)	
Post-dilatation	33 (50.8)	12 (60.0)	21 (46.7)	0.422
**Post-TAVR Echocardiography**				
EF at discharge after TAVR (%)	56.0 ± 11.6	56.3 ± 11.0	55.8 ± 12.0	0.876
Peak gradient at discharge after TAVR (mmHg)	21.0 ± 10.0	21.0 ± 8.3	21.0 ± 10.8	0.983
Mean gradient at discharge after TAVR (mmHg)	12.2 ± 6.2	12.0 ± 5.3	12.2 ± 6.6	0.873
**Echocardiography at 1–3 months after TAVR**				
EF at 1–3 months after TAVR (%)	60.0 ± 9.3	57.5 ± 9.3	61.2 ± 9.1	0.156
Peak gradient at 1–3 months after TAVR (mmHg)	22.7 ± 12.0	28.2 ± 17.1	20.3 ± 8.0	0.059
Mean gradient at 1–3 months after TAVR (mmHg)	12.9 ± 8.5	16.3 ± 13.0	11.4 ± 4.9	0.120
**Changes in echocardiographic parameters (1–3 months vs. discharge after TAVR)**				
Δ EF (1–3 months post-TAVR − discharge EF)	4.1 ± 9.2	1.2 ± 9.1	5.3 ± 9.0	0.102
Δ peak gradient	1.7 ± 12.1	7.3 ± 16.7	−0.7 ± 8.6	0.055
Δ mean gradient	0.8 ± 8.5	4.3 ± 12.4	−0.8 ± 5.5	0.091
Δ peak gradient ≥ 10 mmHg	8 (12.3)	6 (30.0)	2 (4.4)	0.008
Δ mean gradient ≥ 10 mmHg	4 (6.2)	2 (10.0)	2 (4.4)	0.581
**Changes in echocardiographic parameters at 1–3 months compared with pre-TAVR**				
Δ EF (1–3 months post-TAVR − pre-TAVR)	6.5 ± 10.5	1.3 ± 10.9	8.8 ± 9.5	0.012
No improvement/reduction in EF at 1–3 months compared with pre-TAVR	20 (30.8)	11 (55.0)	9 (20.0)	0.008
**CT analysis at 1 month after TAVR**				
TAVR-to-CT interval (days)	41.6 ± 14.4	41.1 ± 14.7	41.8 ± 14.5	0.868
THV centered within the aortic root	37 (56.9)	8 (40.0)	29 (64.4)	0.103
Circular THV expansion	55 (84.6)	17 (85.0)	38 (84.4)	1.000
Neo-sinus–LCA distance (mm)	12.09 ± 4.42	12.28 ± 4.62	12.01 ± 4.38	0.828
Neo-sinus–RCA distance (mm)	14.28 ± 5.68	15.96 ± 6.28	13.53 ± 5.29	0.087
LCA ostial eccentricity (*n*, %)				
Grade 1–2	36 (55.4)	11 (55.0)	25 (55.6)	0.990
Grade 3–4	29 (44.6)	9 (45.0)	20 (44.4)	
RCA ostial eccentricity (*n*, %)				
Grade 1–2	32 (49.2)	6 (30.0)	26 (57.8)	0.059
Grade 3–4	33 (50.8)	14 (70.0)	19 (42.2)	

**Table 2:** Univariable Analysis of Factors Associated With HALT.

Variables	Variable Values	HALT+	HALT−	Crude OR (95% CI)	*p*
Peak gradient at 1–3 months after TAVR (mmHg)	Continuous; OR per 10 mmHg increment	28.2 ± 17.1	20.3 ± 8.0	1.97 (1.10–3.39)	0.032
Δ Peak gradient (1–3 months) − discharge (mmHg)	Continuous; OR per 10 mmHg increment	7.3 ± 16.7	−0.7 ± 8.6	2.16 (1.00–4.41)	0.042
Δ Peak gradient (1–3 months) − discharge ≥ 10 mmHg	No	14/20 (70.0)	43/45 (95.6)	1.00	0.008
	Yes	6/20 (30.0)	2/45 (4.4)	9.21 (1.67–50.96)	
Δ EF (1–3 months) − preTAVR (%)	Continuous; OR per 5 percentage point increment	1.3 ± 10.9	8.8 ± 9.5	0.66 (0.47–0.90)	0.012
No improvement/reduction in EF at 1–3 months compared with pre-TAVR	Improved EF > 0%	9/20 (45.0)	36/45 (80.0)	1.00	0.008
	No improvement/reduction in EF	11/20 (55.0)	9/45 (20.0)	4.89 (1.56–15.35)	
Hemoglobin at 1 month after TAVR (g/L)	Continuous; OR per 1 g/L increase	137.8 ± 14.0	128.6 ± 16.8	1.04 (1.00–1.08)	0.042
RCA ostial eccentricity grade 3–4	Grade 1–2	6/20 (30.0)	26/45 (57.8)	1.00	0.043
	Grade 3–4	14/20 (70.0)	19/45 (42.2)	3.19 (1.03–9.86)	
Neo-sinus–RCA > 13 mm	No	5/20 (25.0)	25/45 (55.6)	1.00	0.027
	Yes	15/20 (75.0)	20/45 (44.4)	3.75 (1.16–12.09)	
RCA ostial eccentricity grade 3–4 + Neo-sinus–RCA > 13 mm	No	8/20 (40.0)	36/45 (80.0)	1.00	0.002
	Yes	12/20 (60.0)	9/45 (20.0)	6.00 (1.89–19.04)	

**Table 3:** Multivariable Logistic Regression Analysis for HALT.

Variables	Variable Values	HALT+	HALT−	Adjusted OR (95% CI)	*p*
RCA ostial eccentricity grade 3–4 + Neo-sinus − RCA distance > 13 mm	No	8/20 (40.0%)	36/45 (80.0%)	1.00	0.006
	Yes	12/20 (60.0%)	9/45 (20.0%)	5.52 (1.63–18.73)	
No improvement/reduction in EF after TAVR at 1–3 months compared with pre-TAVR	Improved EF > 0%	9/20 (45.0%)	36/45 (80.0%)	1.00	0.018
	No improvement/reduction	11/20 (55.0%)	9/45 (20.0%)	4.44 (1.29–15.29)	

## Data Availability

The data presented in this study are available from the corresponding author upon reasonable request. The data are not publicly available due to privacy concerns and ethical restrictions related to patient confidentiality.

## Supplementary Materials

The following supporting information can be downloaded at: https://www.mdpi.com/article/10.3390/jcm15166219/s1. Figure S1: Patient flow diagram. Figure S2: Post-procedural geometric parameters assessed by multidetector computed tomography (MDCT). Figure S3: Definition of coronary ostial eccentricity. Figure S4: Receiver operating characteristic (ROC) curve for neo-sinus–RCA distance in predicting HALT. Figure S5: Schematic illustration of blood washout within the neo-sinus and its proposed relationship with leaflet thrombosis. Table S1: Incidence and Classification of Leaflet Thrombosis (HALT) on Post-TAVR CT. Table S2: Distribution of HALT According to Valve Leaflet Location. Table S3: Number of Valve Leaflets With HALT. Table S4: Frequency of HALT According to Individual Valve Leaflets. Table S5: Severity of HALT. Table S6: HALT Thickness. Table S7: Characteristics of Reduced Leaflet Motion (RLM). Table S8: Distribution of Leaflets With Reduced Leaflet Motion (RLM). Table S9: Severity of Reduced Leaflet Motion (RLM). Table S10: Baseline clinical and paraclinical characteristics of the study population. Table S11: Univariable Analysis of Factors Associated With HALT. Table S12: Assessment of linearity between continuous predictors and the log odds of HALT.

## Abbreviations

The following abbreviations are used in this manuscript:

BEV	Balloon-Expandable Valve
BMI	Body Mass Index
BSA	Body Surface Area
CI	Confidence Interval
CT	Computed Tomography
CVT	Clinical Valve Thrombosis
HALT	Hypoattenuated Leaflet Thickening
LCA	Left Coronary Artery
LVEF	Left Ventricular Ejection Fraction
MDCT	Multidetector Computed Tomography
OR	Odds Ratio
RCA	Right Coronary Artery
RLM	Reduced Leaflet Motion
SLT	Subclinical Leaflet Thrombosis
STROBE	Strengthening the Reporting of Observational Studies in Epidemiology
STS	Society of Thoracic Surgeons
TAVR	Transcatheter Aortic Valve Replacement
THV	Transcatheter Heart Valve
VARC-3	Valve Academic Research Consortium-3
