# Supplementary material for: Post-TAVR Neo-Sinus Geometry and Impaired Ventricular Recovery Are Associated with Early Leaflet Thrombosis: First MDCT Insights from Vietnam
Source: J Clin Med. 2026 Aug 11;15(16):6219. doi: 10.3390/jcm15166219 (PMC13513041; doi:10.3390/jcm15166219)
Supplement: Supplementary file 1 [file jcm-15-06219-s001.zip › jcm-4453391-supplementary.pdf]

SUPPLEMENTAL MATERIAL

Figure S1: Patient-flow diagram

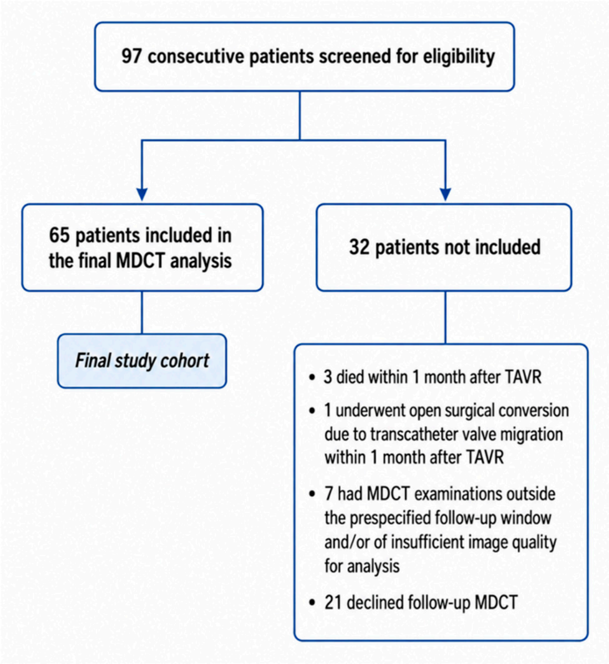

Figure S2: The post-procedural geometric parameters on MDCT.

Schematic illustration of the post-TAVR geometric measurements. The neo-sinus–coronary ostium distance is defined as the vertical distance from the base of the neo-sinus to the coronary ostium. The maximum thrombus thickness is defined as the maximum distance from the base of the neo-sinus to the outer margin of hypoattenuated leaflet thickening (HALT), measured on the vertical plane passing through the opposite THV commissure.

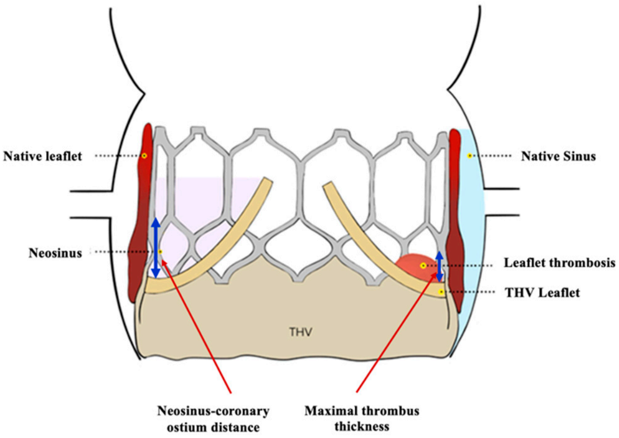

Figure S3: Definition of coronary ostial eccentricity

Coronary ostial eccentricity is the angular deviation of the coronary ostium from the center of the corresponding THV neo-sinus and was classified as centered (0°–10°), mild (10.1°–20°), moderate (20.1°–30°), or severe (>30°).

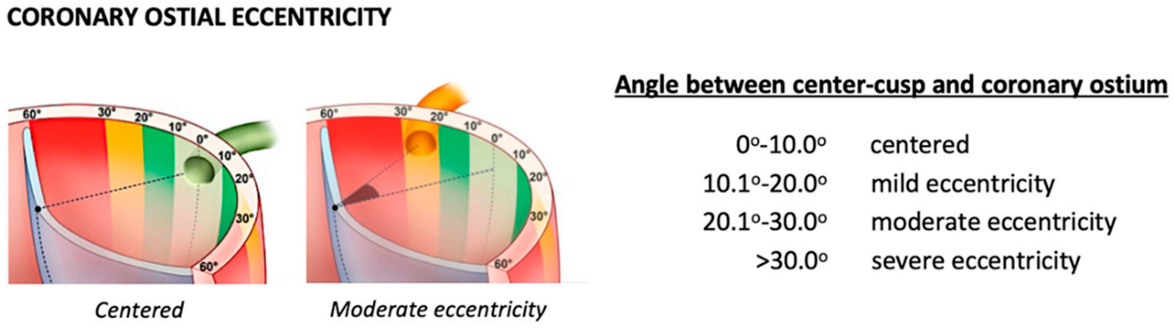

Figure S4: Schematic illustration of blood washout at the base of the neo-sinus and native sinus toward the coronary arteries. A high neo-sinus–RCA distance, combined with severe coronary ostial eccentricity, may impair washout and promote leaflet thrombosis.

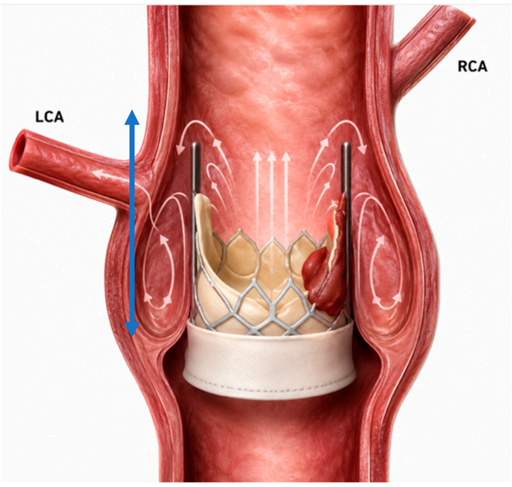

Figure S5: Receiver operating characteristic (ROC) curve for neo-sinus–RCA distance in predicting HALT. The area under the curve (AUC) was 0.634 (95% CI: 0.482–0.787; p = 0.083 vs. AUC = 0.50), indicating modest discrimination. The Youden index identified an optimal cut-off of ≥13.37 mm (sensitivity 75.0%, specificity 57.8%), which classified patients identically to the predefined 13-mm threshold used throughout this study. This threshold should be regarded as exploratory and requires validation in larger, independent cohorts before clinical application.

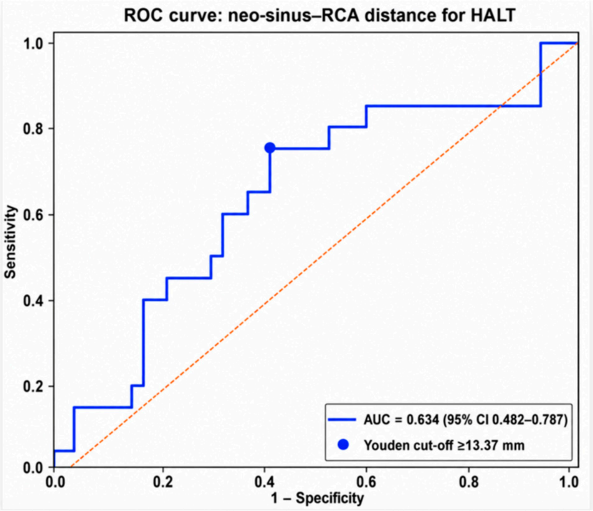

Table S1. Incidence and Classification of Leaflet Thrombosis (HALT) on Post-TAVR CT

Variables	N	Percentage (%)
HALT	20	30.8
SLT	19	29.2
CVT	1	1.5
RLM	16	24.6

Table S2. Distribution of HALT According to Valve Leaflet Location

HALT Location	Number of Cases	Percentage of HALT Cases (n=20)
Left coronary leaflet (L)	4	20.0
Right coronary leaflet (R)	3	15.0
Non-coronary leaflet (N)	4	20.0
Left + right coronary leaflets (L+R)	0	0.0
Left coronary + non-coronary leaflets (L+N)	5	25.0
Right coronary + non-coronary leaflets (R+N)	2	10.0
All three leaflets (L+R+N)	2	10.0
Total HALT cases	20	100.0

Table S3. Number of Valve Leaflets With HALT

Number of Leaflets With HALT	Number of Cases	Percentage of HALT Cases (n=20)
1 leaflet	11	55.0
2 leaflets	7	35.0
3 leaflets	2	10.0

Table S4. Frequency of HALT According to Individual Valve Leaflets

Valve Leaflet Involved in HALT	Number of Cases	Percentage of HALT Cases (n=20)	Percentage of Total HALT Leaflets (n=31)
Left coronary leaflet (L)	11	55.0	35.5
Right coronary leaflet (R)	7	35.0	22.6
Non-coronary leaflet (N)	13	65.0	41.9

Table S5. Severity of HALT

HALT Severity	Number of Cases	Percentage of HALT Cases (n=20)
<25% (Grade 1)	1	5.0
25–50% (Grade 2)	7	35.0
50–75% (Grade 3)	8	40.0
>75% (Grade 4)	4	20.0

Table S6. HALT Thickness

Parameters	Values
Maximum HALT thickness, mm; median (Q1–Q3)	5.21 (4.40–6.32)
Maximum HALT thickness, mm; mean ± SD	5.68 ± 1.99

Table S7. Characteristics of Reduced Leaflet Motion (RLM)

RLM Parameters	Number of Cases/Leaflets	Percentage (%)
RLM in the overall cohort	16	24.6
RLM among HALT+ patients	16	80.0
Number of leaflets with RLM / number of leaflets with HALT	21/31	67.7

Table S8. Distribution of Leaflets With Reduced Leaflet Motion (RLM)

RLM Location	Number of Cases	Percentage of RLM Cases (n=16)
Left coronary leaflet (L)	4	25.0
Right coronary leaflet (R)	2	12.5
Non-coronary leaflet (N)	6	37.5
Left + right coronary leaflets (L+R)	1	6.2
Left coronary + non-coronary leaflets (L+N)	2	12.5
All three leaflets (L+R+N)	1	6.2
Right coronary + non-coronary leaflets (R+N)	0	0.0

Table S9. Severity of Reduced Leaflet Motion (RLM)

RLM Severity	Number of Cases	Percentage of RLM Cases (n=16)
<25% (Grade 1)	4	25.0
25–50% (Grade 2)	7	43.8
50–75% (Grade 3)	5	31.2
>75% (Grade 4)	0	0.0

Table S10. Baseline clinical and paraclinical characteristics of the study population

Characteristics	Overall (n=65)	HALT+ (n=20)	HALT− (n=45)	p
**Anthropometric measurements**				
Age (years)	74 (68–76)	74 (70–76)	73 (67–75)	0.343
Male sex	46 (70.8)	15 (75.0)	31 (68.9)	0.770
Female sex	19 (29.2)	5 (25.0)	14 (31.1)	0.770
BMI (kg/m²)	22.4 (20.6–24.4)	20.9 (19.9–23.1)	22.7 (21.1–24.5)	0.088
BSA (m²)	1.65 (1.49–1.71)	1.61 (1.48–1.69)	1.66 (1.51–1.73)	0.313
**History**				
Type 2 diabetes mellitus	16 (24.6)	6 (30.0)	10 (22.2)	0.542
Hypertension	29 (44.6)	7 (35.0)	22 (48.9)	0.418
Dyslipidemia	10 (15.4)	2 (10.0)	8 (17.8)	0.711
Smoking	19 (29.2)	7 (35.0)	12 (26.7)	0.560
Coronary artery disease	12 (18.5)	5 (25.0)	7 (15.6)	0.490
Previous PCI	7 (10.8)	2 (10.0)	5 (11.1)	1.000
Previous CABG	2 (3.1)	0 (0.0)	2 (4.4)	1.000
Prior stroke	4 (6.2)	0 (0.0)	4 (8.9)	0.303
Peripheral artery disease	2 (3.1)	1 (5.0)	1 (2.2)	0.524
Chronic lung disease	6 (9.2)	2 (10.0)	4 (8.9)	1.000
Previous cardiac valve surgery	5 (7.7)	1 (5.0)	4 (8.9)	1.000
**Surgical risk**				
EuroSCORE II (%)	2.53 (1.42–5.33)	2.05 (1.35–4.78)	2.54 (1.44–5.33)	0.434
STS score (%)	2.63 (1.66–4.63)	2.97 (1.85–5.89)	2.56 (1.58–4.40)	0.345
STS score ≥8%	8 (12.3)	4 (20.0)	4 (8.9)	0.238
**Pre-TAVR echocardiography**				

Pre-TAVR EF (%)	53.5 ± 13.5	56.2 ± 13.1	52.3 ± 13.7	0.283
Aortic valve area (cm²)	0.6 ± 0.2	0.6 ± 0.1	0.5 ± 0.2	0.598
Pre-TAVR peak gradient (mmHg)	87.2 ± 23.3	89.9 ± 19.7	86.1 ± 24.9	0.508
Pre-TAVR mean gradient (mmHg)	55.5 ± 15.8	56.9 ± 13.4	54.9 ± 16.9	0.612
Pre-TAVR Vmax (m/s)	4.6 ± 0.7	4.7 ± 0.7	4.6 ± 0.7	0.428
Mitral regurgitation before TAVR				
Grade 0–4, mean ± SD	1.5 ± 0.9	1.2 ± 0.6	1.6 ± 0.9	0.065
Grade 1–2	56 (86.2)	19 (95.0)	37 (82.2)	0.255
Grade 3–4	8 (12.3)	1 (5.0)	7 (15.6)	0.417
Aortic regurgitation before TAVR				
Grade 0–4, mean ± SD	1.5 ± 0.9	1.7 ± 0.8	1.4 ± 0.9	0.367
Grade 1–2	55 (84.6)	18 (90.0)	37 (82.2)	0.711
Grade 3–4	7 (10.8)	2 (10.0)	5 (11.1)	1.000
Pre-TAVR pulmonary artery pressure (mmHg)	38.2 ± 11.0	36.4 ± 8.5	39.0 ± 11.9	0.317
Ascending aorta diameter on pre-TAVR echocardiography (mm)	36.1 ± 5.0	36.4 ± 4.0	36.0 ± 5.4	0.746
**Aortic stenosis stage**				
Stage 1	59 (90.8)	20 (100.0)	39 (86.7)	0.230
Stage 2	3 (4.6)	0 (0.0)	3 (6.7)	
Stage 3	3 (4.6)	0 (0.0)	3 (6.7)	
**Pre-TAVR laboratory findings**				
Hemoglobin before TAVR (g/L)	135.6 ± 15.9	137.8 ± 12.3	134.6 ± 17.2	0.393
White blood cell count before TAVR (G/L)	7.9 ± 2.3	8.2 ± 1.9	7.8 ± 2.4	0.531
Platelet count before TAVR (G/L)	211.5 ± 76.3	205.7 ± 108.9	214.1 ± 57.7	0.749
Creatinine before TAVR (μmol/L)	95.3 ± 32.3	97.8 ± 26.7	94.2 ± 34.8	0.642
GFR before TAVR (mL/min/1.73m²)	65.0 ± 21.2	62.5 ± 20.2	66.2 ± 21.8	0.513
Blood group				
Group A	18 (27.7)	6 (30.0)	12 (26.7)	0.663
Group B	19 (29.2)	7 (35.0)	12 (26.7)	
Group O	28 (43.1)	7 (35.0)	21 (46.7)	
**Pre-TAVR MDCT characteristics**				
Tricuspid aortic valve	23 (35.4)	9 (45.0)	14 (31.1)	0.530
Bicuspid aortic valve	37 (56.9)	10 (50.0)	27 (60.0)	
Biological valve	5 (7.7)	1 (5.0)	4 (8.9)	
Annular area (mm²)	438.7 ± 113.4	445.2 ± 107.4	435.8 ± 117.0	0.754
Annular perimeter (mm)	74.8 ± 9.7	75.4 ± 9.2	74.6 ± 10.1	0.735
LVOT area (mm²)	458.9 ± 119.7	461.0 ± 117.5	457.9 ± 121.9	0.925
Mean sinus of Valsalva diameter (mm)	31.6 ± 3.7	32.6 ± 2.9	31.2 ± 4.0	0.110
Mean STJ diameter (mm)	29.8 ± 4.0	30.8 ± 3.8	29.4 ± 4.1	0.203
Left coronary artery height (mm)	14.1 ± 3.4	14.4 ± 2.5	14.0 ± 3.7	0.596
Right coronary artery height (mm)	16.4 ± 4.5	17.0 ± 5.9	16.1 ± 3.7	0.511
Annulus angle (degrees)	49.2 ± 9.9	51.2 ± 10.4	48.4 ± 9.7	0.305
Maximum ascending aorta diameter (mm)	38.3 ± 5.2	38.9 ± 4.8	38.1 ± 5.4	0.518
Calcification score (Agatston)	4136.8 ± 2425.4	4827.6 ± 2118.3	3829.8 ± 2524.6	0.126
Severe calcification	46 (70.8%)	15 (75.0%)	31 (68.9%)	0.770

**TAVR procedure**				
Pre-dilatation	58 (89.2)	19 (95.0)	39 (86.7)	0.423
Balloon-expandable valve	49 (75.4)	17 (85.0)	32 (71.1)	0.375
Self-expandable valve	16 (24.6)	3 (15.0)	13 (28.9)	
Small THV size (20–23 mm)	30 (46.2)	10 (50.0)	20 (44.4)	0.410
Medium THV size (24.5–27.5 mm)	20 (30.8)	4 (20.0)	16 (35.6)	
Large THV size (29–30.5 mm)	15 (23.1)	6 (30.0)	9 (20.0)	
Post-dilatation	33 (50.8)	12 (60.0)	21 (46.7)	0.422
**Post-TAVR Echocardiography**				
EF at discharge after TAVR (%)	56.0 ± 11.6	56.3 ± 11.0	55.8 ± 12.0	0.876
Peak gradient at discharge after TAVR (mmHg)	21.0 ± 10.0	21.0 ± 8.3	21.0 ± 10.8	0.983
Mean gradient at discharge after TAVR (mmHg)	12.2 ± 6.2	12.0 ± 5.3	12.2 ± 6.6	0.873
Mitral regurgitation at discharge after TAVR				
Grade 0–4, mean ± SD	1.2 ± 0.7	1.1 ± 0.4	1.3 ± 0.8	0.109
Grade 1–2	57 (87.7)	19 (95.0)	38 (84.4)	0.417
Grade 3–4	3 (4.6)	0 (0.0)	3 (6.7)	0.547
Aortic regurgitation at discharge after TAVR				
Grade 0–4, mean ± SD	1.0 ± 0.2	0.9 ± 0.2	1.1 ± 0.3	0.069
Grade 1–2	64 (98.5)	19 (95.0)	45 (100.0)	0.308
Grade 3–4	0 (0.0)	0 (0.0)	0 (0.0)	—
Pulmonary artery pressure at discharge after TAVR (mmHg)	32.6 ± 6.6	33.0 ± 5.5	32.4 ± 7.1	0.707
**Changes in echocardiographic parameters (before TAVR vs discharge)**				
Δ EF (discharge – pre-TAVR)	2.4 ± 7.5	0.1 ± 7.1	3.5 ± 7.5	0.087
**Laboratory findings after TAVR**				
Hemoglobin after TAVR (g/L)	118.7 ± 21.3	121.7 ± 20.7	117.3 ± 21.6	0.444
White blood cell count after TAVR (G/L)	11.3 ± 5.1	10.5 ± 4.2	11.7 ± 5.5	0.366
Platelet count after TAVR (G/L)	180.3 ± 78.3	177.1 ± 85.6	181.7 ± 75.7	0.838
Creatinine after TAVR (µmol/L)	93.0 ± 42.5	88.2 ± 26.8	95.1 ± 47.9	0.460
GFR after TAVR (mL/min/1.73m²)	64.7 ± 22.0	65.3 ± 21.2	64.4 ± 22.6	0.880
**Echocardiography at 1–3 months after TAVR**				
EF at 1–3 months after TAVR (%)	60.0 ± 9.3	57.5 ± 9.3	61.2 ± 9.1	0.156
Peak gradient at 1–3 months after TAVR (mmHg)	22.7 ± 12.0	28.2 ± 17.1	20.3 ± 8.0	0.059
Mean gradient at 1–3 months after TAVR (mmHg)	12.9 ± 8.5	16.3 ± 13.0	11.4 ± 4.9	0.120
Mitral regurgitation at 1–3 months after TAVR				
Grade 0–4, mean ± SD	1.0 ± 0.3	1.0 ± 0.3	1.0 ± 0.3	0.789
Grade 1–2	53 (81.5)	18 (90.0)	35 (77.8)	0.315
Grade 3–4	4 (6.2)	0 (0.0)	4 (8.9)	0.303
Aortic regurgitation at 1–3 months after TAVR				
Grade 0–4, mean ± SD	1.0 ± 0.3	1.0 ± 0.3	1.0 ± 0.3	0.789
Grade 1–2	62 (95.4)	19 (95.0)	43 (95.6)	1.000
Grade 3–4	0 (0.0)	0 (0.0)	0 (0.0)	
Pulmonary artery pressure at 1–3 months after TAVR (mmHg)	32.0 ± 5.9	30.2 ± 5.7	32.7 ± 5.9	0.108

**Changes in echocardiographic parameters (1-3 months vs discharge after TAVR)**				
Δ EF (1-3 months post-TAVR – discharge EF)	4.1 ± 9.2	1.2 ± 9.1	5.3 ± 9.0	0.102
Δ peak gradient	1.7 ± 12.1	7.3 ± 16.7	−0.7 ± 8.6	0.055
Δ mean gradient	0.8 ± 8.5	4.3 ± 12.4	−0.8 ± 5.5	0.091
Δ peak gradient ≥10 mmHg	8 (12.3)	6 (30.0)	2 (4.4)	0.008
Δ mean gradient ≥10 mmHg	4 (6.2)	2 (10.0)	2 (4.4)	0.581
**Changes in echocardiographic parameters at 1–3 months compared with pre-TAVR**				
Δ EF (1–3 months post-TAVR – pre-TAVR)	6.5 ± 10.5	1.3 ± 10.9	8.8 ± 9.5	0.012
No improvement/reduction in EF at 1–3 months compared with pre-TAVR	20 (30.8)	11 (55.0)	9 (20.0)	0.008
**Laboratory findings at 1 month after TAVR**				
Hemoglobin at 1 month after TAVR (g/L)	131.4 ± 16.5	137.8 ± 14.0	128.6 ± 16.8	0.026
White blood cell count at 1 month after TAVR (G/L)	7.9 ± 2.3	7.9 ± 2.9	7.9 ± 2.1	0.990
Platelet count at 1 month after TAVR (G/L)	212.2 ± 70.5	194.8 ± 76.4	219.8 ± 67.1	0.216
Creatinine at 1 month after TAVR (μmol/L)	91.9 ± 32.7	88.2 ± 25.8	93.5 ± 35.4	0.498
GFR at 1 month after TAVR (mL/min/1.73m²)	66.7 ± 21.2	67.9 ± 20.6	66.1 ± 21.6	0.754
**Medications at discharge**				
Antithrombotic therapy at discharge				
Oral anticoagulation (VKA or DOAC)	5 (7.7%)	1 (5%)	5 (11.1%)	0.573
Antiplatelet therapy (DAPT or SAPT)	58 (89.2%)	19 (95%)	39 (86.7%)	
No Antithrombotic therapy	1 (1.5%)	0 (0%)	1 (2.2%)	
ACEi/ARNI	38 (58.5)	9 (45.0)	29 (64.4)	0.177
Beta-blockers	26 (40.0)	8 (40.0)	18 (40.0)	1.000
Aldosterone antagonists	28 (43.1)	5 (25.0)	23 (51.1)	0.061
SGLT2 inhibitors	52 (80.0)	16 (80.0)	36 (80.0)	1.000
Loop diuretics	21 (32.3)	3 (15.0)	18 (40.0)	0.083
Statins	57 (87.7)	17 (85.0)	40 (88.9)	0.693
**CT analysis at 1 month after TAVR**				
TAVR-to-CT interval (days)	41.6 ± 14.4	41.1 ± 14.7	41.8 ± 14.5	0.868
THV centered within the aortic root	37 (56.9)	8 (40.0)	29 (64.4)	0.103
Circular THV expansion	55 (84.6)	17 (85.0)	38 (84.4)	1.000
Symmetric longitudinal BEV expansion (N=49)	41 (83.7%)	15 (88.2%)	26 (81.2%)	0.699
Neo-sinus–LCA distance (mm)	12.09 ± 4.42	12.28 ± 4.62	12.01 ± 4.38	0.828
Neo-sinus–RCA distance (mm)	14.28 ± 5.68	15.96 ± 6.28	13.53 ± 5.29	0.087
LCA ostial eccentricity (n, %)				
Grade 1–2	36 (55.4)	11 (55.0)	25 (55.6)	0.990
Grade 3–4	29 (44.6)	9 (45.0)	20 (44.4)	
RCA ostial eccentricity (n, %)				
Grade 1–2	32 (49.2%)	6 (30.0%)	26 (57.8%)	0.059
Grade 3–4	33 (50.8%)	14 (70.0%)	19 (42.2%)	

Table S11. Univariable Analysis of Factors Associated With HALT

Variables	Variable Values	HALT+	HALT−	Crude OR (95% CI)	p
Peak gradient at 1–3 months after TAVR (mmHg)	Continuous; OR per 10-mmHg increment	28.2 ± 17.1	20.3 ± 8.0	1.97 (1.10–3.39)	0.032
Δ Peak gradient (1–3 months) – discharge (mmHg)	Continuous; OR per 10-mmHg increment	7.3 ± 16.7	−0.7 ± 8.6	2.16 (1.00–4.41)	0.042
Δ Mean gradient (1–3 months) – discharge (mmHg)	Continuous; OR per 1 mmHg increase	4.3 ± 12.4	−0.8 ± 5.5	1.11 (0.99–1.24)	0.073
Δ Mean gradient (1–3 months) – discharge ≥10 mmHg	No	18/20 (90.0)	43/45 (95.6)	1.00	0.581
	Yes	2/20 (10.0)	2/45 (4.4)	2.39 (0.31–18.29)	
Δ Peak gradient (1–3 months) – discharge ≥10 mmHg	No	14/20 (70.0)	43/45 (95.6)	1.00	0.008
	Yes	6/20 (30.0)	2/45 (4.4)	9.21 (1.67–50.96)	
Δ EF discharge – preTAVR (%)	Continuous; OR per 1% increase	0.1 ± 7.1	3.5 ± 7.5	0.94 (0.87–1.01)	0.096
Δ EF (1–3 months) – preTAVR (%)	Continuous; OR per 5 percentage point increment	1.3 ± 10.9	8.8 ± 9.5	0.66 (0.47–0.90)	0.012
No improvement/reduction in EF at 1–3 months compared with pre-TAVR	Improved EF >0%	9/20 (45.0)	36/45 (80.0)	1.00	0.008
	No improvement/reduction in EF	11/20 (55.0)	9/45 (20.0)	4.89 (1.56–15.35)	
Hemoglobin at 1 month after TAVR (g/L)	Continuous; OR per 1 g/L increase	137.8 ± 14.0	128.6 ± 16.8	1.04 (1.00–1.08)	0.042
RCA ostial eccentricity grade 3–4	Grade 1–2	6/20 (30.0)	26/45 (57.8)	1.00	0.043
	Grade 3–4	14/20 (70.0)	19/45 (42.2)	3.19 (1.03–9.86)	
Neo-sinus–RCA >12 mm	No	5/20 (25.0)	21/45 (46.7)	1.00	0.106
	Yes	15/20 (75.0)	24/45 (53.3)	2.63 (0.82–8.45)	
Neo-sinus–RCA >13 mm	No	5/20 (25.0)	25/45 (55.6)	1.00	0.027
	Yes	15/20 (75.0)	20/45 (44.4)	3.75 (1.16–12.09)	
Neo-sinus–RCA >14 mm	No	7/20 (35.0)	26/45 (57.8)	1.00	0.094
	Yes	13/20 (65.0)	19/45 (42.2)	2.54 (0.85–7.58)	
	No	9/20 (45.0)	30/45 (66.7)	1.00	

Neo-sinus–RCA >15 mm	Yes	11/20 (55.0)	15/45 (33.3)	2.44 (0.83–7.18)	0.104
Neo-sinus–RCA >16 mm	No	11/20 (55.0)	34/45 (75.6)	1.00	0.102
	Yes	9/20 (45.0)	11/45 (24.4)	2.53 (0.83–7.70)	
RCA ostial eccentricity grade 3–4 + Neo-sinus–RCA >13 mm	No	8/20 (40.0)	36/45 (80.0)	1.00	0.002
	Yes	12/20 (60.0)	9/45 (20.0)	6.00 (1.89–19.04)	
THV centered within the aortic root	No	12/20 (60.0)	16/45 (35.6)	1.00	0.103
	Yes	8/20 (40.0)	29/45 (64.4)	0.37 (0.12–1.09)	
BEV symmetric longitudinal expansion (n=49)	No	5/15 (33.3)	11/34 (32.4)	1.00	1.000
	Yes	10/15 (66.7)	23/34 (67.6)	0.96 (0.25–3.63)	
Circular THV expansion	No	3/20 (15.0)	7/45 (15.6)	1.00	1.000
	Yes	17/20 (85.0)	38/45 (84.4)	1.04 (0.24–4.53)	

Table S12: Assessment of linearity between continuous predictors and the log odds of HALT

Continuous predictor	n	Univariable OR (95% CI)	P value	Box–Tidwell P for nonlinearity	Restricted cubic spline P for nonlinearity
Peak gradient at 1–3 months after TAVR (mmHg)	65	Continuous; OR per 10-mmHg increment	0.032	0.463	0.334
Δ Peak gradient (1–3 months) – discharge (mmHg)	65	Continuous; OR per 10-mmHg increment	0.042	0.971	0.851
Δ EF (1–3 months) – preTAVR (%)	65	0.92 (0.86–0.98)	0.012	0.117	0.083
Hemoglobin at 1 month after TAVR (g/L)	65	1.04 (1.00–1.08)	0.042	0.767	0.785
Neo-sinus–RCA distance	65	1.08 (0.98–1.19)	0.122	0.861	0.783

Footnote: ORs were obtained from univariable logistic regression models. The linearity of continuous predictors with the log odds of HALT was assessed using the Box–Tidwell procedure and further evaluated using restricted cubic spline models. Restricted cubic spline models were compared with corresponding linear logistic regression models using likelihood-ratio tests. No statistically significant departures from linearity were detected for any continuous predictor.
